# Access to cardiac rehabilitation and the role of language barriers in the provision of cardiac rehabilitation to migrants

**DOI:** 10.1186/s12913-019-4041-1

**Published:** 2019-04-11

**Authors:** Fatima Al-Sharifi, Hanne Winther Frederiksen, Henriette Knold Rossau, Marie Norredam, Ann-Dorthe Zwisler

**Affiliations:** 1 Section of Immigrant Medicine, Department of Infectious Disease, Copenhagen University Hospital, Ostvej, Pavillion 2, 2650 Hvidovre, Copenhagen, Denmark; 2 Knowledge Centre for Rehabilitation and Palliative Care (REHPA) Odense University Hospital and University of Southern Denmark, Odense, Denmark; 3 Research Centre for Migration, Ethnicity and Health (MESU), Section of Health Services Research, University of Copenhagen, Copenhagen, Denmark; 4 Department of Internal Medicine, Copenhagen University Hospital, Herlev, Denmark

**Keywords:** Cardiac rehabilitation, Migrants, Translations, Language barriers

## Abstract

**Background:**

Cardiac rehabilitation (CR) has proven health benefits and, according to international guidelines, CR must be offered to all eligible patients. Studies have reported lower uptake of CR among migrants, and migrants are known to face several barriers in their access to healthcare, of which language is the most common. The aim of this study is to examine the provision of CR core components for migrants; and the role of language barriers in the provision of CR in Danish hospitals and municipalities.

**Methods:**

This is a descriptive study based on repeated nationwide surveys conducted in 2013 and 2015 by the Danish Cardiac Rehabilitation Database. The surveys collected information on provision and organization of CR in hospitals (*n* = 35) and municipalities (*n* = 98) in Denmark. The survey in 2015 had additional items related to migrants, such as provision of interpreter services and multilingual information material.

**Results:**

Not all CR core components were provided by hospitals to non-Danish speaking patients. There was no improvement from 2013 to 2015. Hospitals had full coverage (19/19) of interpreter services compared to 84% (26/31) of municipalities. Provision of multilingual information material was low in hospitals 32% (6/19) and in municipalities 3% (1/31).

**Conclusion:**

This study found language-related barriers in migrants’ access to CR, in the form of inadequate provision of CR core components for non-Danish speaking patients at some Danish hospitals and suboptimal provision of interpreter services in municipalities. The findings call for increased attention to language barriers and further studies are needed to map the extent of the problem.

**Electronic supplementary material:**

The online version of this article (10.1186/s12913-019-4041-1) contains supplementary material, which is available to authorized users.

## Background

Ischemic heart disease (IHD) ranks as the leading cause of mortality and morbidity globally [1]. There is robust evidence that participation in cardiac rehabilitation (CR) following a cardiac event reduces IHD mortality, morbidity and rehospitalisation in addition to improving quality of life [2, 3]. Correspondingly, CR is recommended in several national and international guidelines [4, 5] including the Danish national clinical guideline for CR [6], which entitles heart patients to CR in hospitals and in municipalities.

Provision of outpatient CR in Denmark was solely a hospital-based task until 2007. In accordance with the World Health Organization’s (WHO) recommendations [7, 8]; a Danish structural reform led to the delivery of CR becoming a shared responsibility between hospitals and municipalities [9]. Community-based outpatient services are thought to enhance accessibility by increasing proximity to the provider and thereby potentially diminishing health inequalities among vulnerable population groups. Vulnerability can relate to socioeconomic status, educational level, age, gender or ethnic origin, and have been shown to predict lower uptake of CR [10–12]. A Danish study in 2012 tested an intervention where socially vulnerable groups were offered an extended rehabilitation programme customized to meet their special needs. Non-Danish speaking patients were included in the study and were offered interpretation support. The study suggested that offering socially differentiated CR can equalize attendance and adherence among socially vulnerable groups [13].

A recent Danish nationwide register study has demonstrated major differences in uptake of hospital-based CR-services between migrants and Danish born patients in the form of fewer contacts for physical exercise and lower initiation of patient education, as well as lower uptake of pharmacological secondary prevention. These findings could not be explained by comorbidity and sociodemographic factors [14]. Other studies have found similar tendencies in uptake of CR among migrants [12, 15, 16].

Migrants are known to face various barriers in their access to healthcare on an individual level in the form of language, culture, inadequate health literacy and difficulties in navigating the healthcare system [17]. Additionally, barriers can also be found on a structural level including the referral system, limited provision of interpreter services, lack of translated and customized information materials for ethnic minority subgroups, short consultation times and resource constraints [18, 19].

Access to healthcare services is defined as the opportunity or ease with which users are able to reach and obtain appropriate services in proportion with their needs. The accessibility of a healthcare service is influenced by factors on an individual level as well as provider and system level [20]. WHO has developed a framework called ‘the right to health framework’ pointing out four indicators to ensure equality in healthcare, these indicators are *accessibility, availability, acceptability* and *quality of service* [21].

Language barriers are among the most common obstacles migrants endure in their access to healthcare, as it can potentially impede effective communication and create misunderstandings, thus posing challenges to providing adequate healthcare. Several international studies have concluded that language barriers hamper access and reduce quality of healthcare [22–24]. However, the use of professional interpreters with non-Danish speaking patients has been associated with improvements in patient satisfaction, clinical outcomes and healthcare access [22, 25, 26].

Additionally, well-designed information material can improve patients’ health knowledge, engagement and patient-doctor communication [27]. A German study from 2016 found that utilization of high-quality multilingual information material was useful among migrants [28]. Hence, the objectives of this study were to examine the provision of core CR components (i.e. exercise training, patient education, nutritional counselling, psychosocial support and smoking cessation support) for migrants and the provision of interpreter services and multilingual materials in hospitals and municipalities.

## Methods

### Study design and setting

The present study is a survey-based descriptive study. Data on the provision of CR services in hospitals and municipalities in Denmark are based on nationwide surveys conducted by the Danish Cardiac Rehabilitation Database (DHRD) [29] in 2013 and 2015. The Danish healthcare system is publicly financed through taxes and operates across three political and administrative levels, national, regional and local. The state has a regulatory and supervisory function. The state is divided into five regions that are responsible for hospitals, general practitioners and psychiatric care. The regions contain 98 municipalities that are responsible for providing primary healthcare services [30].

### Data collection and respondents

Information on core components of available cardiac rehabilitation programmes and organization of CR services were collected by way of parallel web-based questionnaires sent out to all hospitals offering CR services (*n* = 35) and all Danish municipalities (*n* = 98). Core components of CR included exercise training, patient education, psychosocial support, smoking cessation support and nutritional counselling.

The respondents were physicians, nurses, dietitians and physiotherapists, who are part of the multidisciplinary CR team. Each of them had to respond to a section of the questionnaire related to their profession.

The questionnaires were complied using the recommended national clinical guideline regarding the coverage of core CR components. A more detailed description of the survey can be found elsewhere [31]. Hospital-based questionnaires were derived from the DHRD, which collects programme-level CR data. The submission of data is required for hospitals according to Danish Law whereas the participation for the municipalities is optional. A combination of closed-ended and open-ended questions was applied to give respondents the opportunity to elaborate on their answers.

In the present study we used hospital data from 2013 and 2015 to examine provision of CR core components for migrants and data from 2015 to examine the distribution of provision across the five Danish regions, and whether hospital type and percentage of migrants in a hospital’s catchment area was related to provision.

The survey from 2015 contained additional questions about socially differentiated CR, socially vulnerable groups including migrants and special offers to these groups. Due to survey design, data on provision of interpreter services and multilingual information material was only obtained from hospitals and municipalities that offer socially differentiated CR.

Finally, we investigated factors that could potentially predict the provision of interpreter services and multilingual material. For hospitals, data variables applied were hospital type and percentage of migrants in a hospital’s catchment area. Municipality data variables were percentage of migrants and size of population in the municipality.

### Definitions

#### Migrants

Migrants are defined by UNESCO as “any person who lives temporarily or permanently in a country where he or she was not born, and has acquired some significant social ties to this country’”.

The survey used in this study was not designed to specifically ask about provision of CR to migrants, however based on the UNESCO definition of migrants and Denmark’s recent immigration history, anyone in Denmark with limited proficiency in Danish or from an ethnic minority background is likely to be a migrant. Hence, migrant involvement in CR was extrapolated from a positive answer to any one of three questions: 1) provision of CR to patients with limited Danish proficiency; 2) provision of CR to non-Danish speaking patients or 3) where CR was provided to patients identified as ethnic minorities.

#### Socially differentiated CR

Socially differentiated CR was not specifically defined in the questionnaire. It was stated that:“ *… patients, who are in need of socially differentiated CR, can often be considered vulnerable or at risk of low uptake of CR”.*

#### Hospital type

In 2015, all of the 35 Danish acute care hospitals offered CR. Of those, 18 were university hospitals and 17 were general hospitals.

##### Data analysis

We applied descriptive statistical analysis. To investigate predictors for provision of CR core components, provision of interpreter services and multilingual information material, we dichotomized hospitals into hospitals with low and high proportion of migrants in their catchment area at a cut-off point of 4% to form two nearly equal-sized groups and according to hospital type (university hospital and general hospital).

Municipalities were dichotomized according to the proportion of migrants in the municipality at a cut-off point of 3,5% to form two nearly equal-sized groups, and according to population size (< 45.000 and > 45.000).

Fisher’s exact test with a significance level of 0.05 was applied to test statistically significant differences between hospitals and municipalities and predictions. Predictors for hospitals’ provision of interpreter services were not analysed due to 100% coverage of interpreter services in all hospitals. Statistical analyses were performed with SAS 9.4 statistical software (SAS Institute Inc., Cary, USA).

## Results

The following core components were included in the surveys in 2013 and 2015: exercise training, patient education, nutritional counselling, psychosocial support and smoking cessation support.

Only the survey from 2015 contained questions about socially differentiated CR and data on provision of interpreter services and multilingual information was only possible to obtain from hospitals and municipalities offering socially differentiated CR due to survey design. In contrast, the 2013 survey did not contain questions on socially differentiated CR and the provision of interpreter services and multilingual information material was included as general questions to all hospitals and municipalities.

### Response rates and participation

**Fig. 1:**
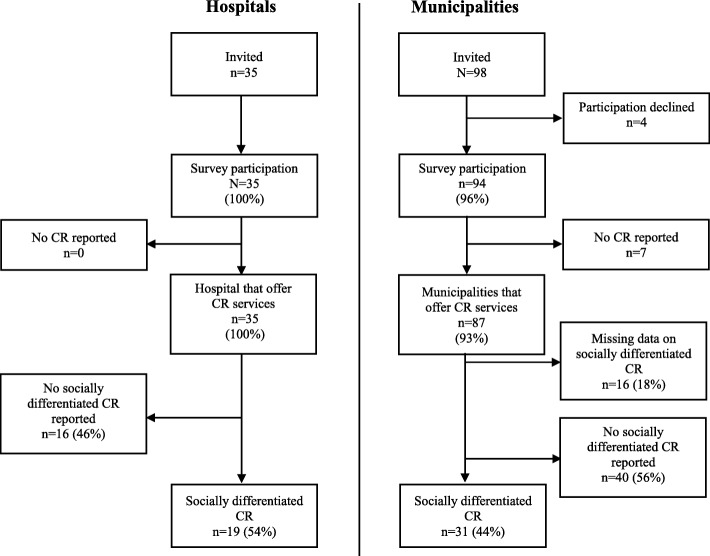
Shows the participation flowchart in survey on provision of CR in hospitals and municipalities 2015

Response rates for hospitals reached 100 and 96% for municipalities, of which 100 and 93% responded that they offer at least one CR core component (Fig. 1).

### Provision of core CR components to migrants

The study found incomplete provision of CR core components to migrants in hospitals. These provision rates were virtually unchanged from 2013 to 2015 (Additional file 1: Table S1). Moreover, in 2015 the provision of each CR core component to migrants in the five Danish regions ranged from 44 to 100% and none of the regions had full provision of all core components in general (Table 1).

**Table 1:** Provision of CR core components for non-Danish speaking patients by different variables in 2015

	Exercise training	Patient education	Psychosocial support	Smoking cessation support	Nutritional counselling
% (n/N)					
Regions					
Capital Region of Denmark	78% (7/9)	44% (4/9)	100% (8/8)	78% (7/9)	89% (8/9)
Central Denmark Region	100% (7/7)	100% (6/6)	100% (6/6)	67% (4/6)	100% (6/6)
North Denmark Region	75% (3/4)	75% (3/4)	50% (2/4)	50% (2/4)	75% (3/4)
Region Zealand	100% (6/6)	83% (5/6)	100% (5/5)	50% (3/6)	100% (6/6)
Region of Southern Denmark	100% (8/8)	78% (7/9)	100% (8/8)	100% (7/7)	100% (8/8)
Hospital type					
University hospital	94% (17/18)	59% (10/17)	94% (15/16)	64% (11/17)	88% (15/17)
General hospital	88% (14/16)	88% (15/17)	93% (14/15)	80% (12/15)	100% (16/16)
Percentage of migrants in hospitals catchment area					
< 4%	90% (18/20)	80% (16/20)	94% (17/18)	74% (14/19)	100% (18/18)
> 4%	93% (13/14)	64% (9/14)	92% (12/13)	69% (9/13)	87% (13/15)

*n* = number of hospitals offering CR core component to migrants

*N* = total number of hospitals offering the CR core component overall

### Socially differentiated CR and vulnerable patient groups

Socially differentiated CR was offered in 54% (*n* = 19/35) of the hospitals, and in 44% (*n* = 31/71) of the municipalities. Overall migrants were considered vulnerable in up to 84% of the hospitals, more specifically 74% were non-Danish speaking patients and 84% were patients with an ethnic minority background (see definition of migrants in this study in the methods section). The corresponding percentages for municipalities were respectively 58 and 61%. These proportions did not differ considerably in percentage terms from other potentially vulnerable groups such as patients with low education, cognitive disability and those living alone except from those with a long distance to provider. No statistical comparison measure was calculated between groups. There were no significant differences between hospitals and municipalities (Table 2).

**Table 2:** Socially differentiated CR, provision of interpreter services, multilingual information material and vulnerable patients in 2015

	Hospitals *N* = 35	Municipalities *N* = 87	*p*-value
Provide socially differentiated cardiac rehabilitation	54% (19/35)	44% (31/71)	0.4082
Provision of interpreter services during rehabilitation	100% (19/19)	84% (26/31)	0.1424
Provision of multilingual information material	32% (6/19)	3% (1/31)	0.009
Patients considered vulnerable			
Low education level	84% (16/19)	65% (20/31)	0.1972
Cognitive disability	84% (16/19)	74% (23/31)	0.4979
Ethnic minority background	74% (14/19)	58% (18/31)	0.3659
Non-Danish speaking patients	84% (16/19)	61% (19/31)	0.1171
Living alone	84% (16/19)	65% (20/31)	0.1972
Limited social network	89% (17/19)	71% (22/31)	0.1699
Multimorbidity	79% (15/19)	77% (24/31)	1.00
Long distance to provider	47% (9/19)	29% (9/31)	0.2331
Psychiatric disorder	84% (16/19)	68% (21/31)	0.3203
Alcohol or drug abuse	84% (16/19)	65% (20/31)	0.1972

### Interpreter services and information material

Provision of interpreter services in hospitals reached full coverage, whereas it was lower in percentage terms in municipalities 84% (*n* = 26/31), the difference was not statistically significant. The availability of multilingual information material was low in hospitals 32% (*n* = 6/19) and in municipalities 3% (*n* = 1/31). The difference between hospitals and municipalities was significant (*p* = 0.009) (Table 2).

University hospitals had a higher provision of multilingual information material 44% (*n* = 4/9) than general hospitals 20% (*n* = 2/10), and hospitals with the highest proportions of migrants in their catchment area were more likely to have multilingual information material 67% (*n* = 6/10), although numbers were small. The association between provision of multilingual information material and proportion of migrants in a hospital’s catchment area was significant (*p* = 0.003).

Provision of interpreter services was not associated with the proportion of migrants. Municipalities with the largest populations were more likely to offer interpreter services 90% (*n* = 19/21) than the ones with smaller populations 70% (*n* = 7/10) but the difference was not significant (Table 3).

**Table 3:** Predictors of provision of interpreter services in CR at hospitals and in municipalities in 2015

	Provision of interpreter services	*p*-value	Multilingual information material	*p*-value
Hospitals				
Hospital type	*	*		0.3498
University Hospital			44% (4/9)	
General Hospital			20% (2/10)	
Percentage of migrants in hospitals’ catchment area				0.0031
< 4%	*	*	0% (0/9)	
> 4%			67% (6/10)	
Municipalities				
Percentage of migrants in municipalities		1.00	**	**
< 3.5% *(n = 42)*	83% (15/18)			
> 3.5% *(n = 45)*	85% (11/13)			
Population size in municipalities		0.2955	**	**
< 45.000 *(n = 45)*	70% (7/10)			
> 45.000 *(n = 42)*	90% (19/21)			

* *p* = 1.00

** Not analyzed due to small number (*n* = 1)

### Open-ended questions

Some hospital respondents commented in the open-ended questions that they specifically referred migrants to the municipality where there are customized offers for non-Danish speaking patients.

Open ended responses also revealed that some respondents did not respond to consider migrants as vulnerable in the close-ended questions because they did not want to stereotype patients, but instead performed an individualized assessment of the patients’ needs.

## Discussion

Our study showed that not all hospitals provided non-Danish speaking patients with all CR core components, and no improvement was seen from 2013 to 2015. Hospitals in Denmark had full coverage of interpreter services, compared to 84% of municipalities. Provision of multilingual information material was low in hospitals (32%) and almost absent in municipalities (3%).

### Provision of CR and access for migrants

According to our results, hospitals did not have full provision of CR core components to non-Danish speaking patients. Only minor changes in provision of CR services were observed from the years 2013 to 2015, e.g. the provision of exercise training increased from 91 to 92%; and the provision of patient education increased from 71 to 74%. These percentages must be interpreted with caution as a few respondents from hospitals explained that they did not exclude non-Danish speaking patients but would refer these patients to municipalities with whom they share responsibility for CR, and therefore had indicated not to provide CR services at the hospital. However, some of the negative responses were left without any comments, and the reasons behind this potential lack of available services needs further investigation. Where language barriers were the reasons given for some hospitals not to provide CR, it would contradict the recommendations of the national clinical guideline for CR [6]. The guideline recommends that CR should be offered to all eligible patients regardless of background and advises providers to be attentive to well-known barriers to participation, such as language. Even though the offer of interpretation is available in theory, this is often organized based on the principal of individual consultation. CR is group based by nature and therefore people with language barriers could be excluded [32]. The right to equal access to healthcare services is also established in Danish law and international human rights covenants [21, 33]. We found a variation in the proportion of hospitals that offered CR to migrants across the five Danish regions; and no region had full provision of core components. A recent Danish study on rehabilitation after brain injury showed regional variations in rehabilitation despite the existence of a national guideline [34]. Together with our findings, this could reflect that the implementation of guidelines can be challenging, and requires monitoring in order to ensure they improve quality in clinical practice [35]. The health system could reduce disparity in healthcare by standardized descriptions of disease management programmes, which are implemented by law, in order that all patients receive treatment of uniform high quality regardless of where they live. This has been proven to be effective in the field of cancer treatment’.

### Vulnerability of migrants and provision of interpretation and multilingual information material

Socially differentiated CR that focused on vulnerable patients was offered in 54% of the hospitals, and in 44% of the municipalities that provided data but, the open-ended answers revealed that there was no uniform understanding of the term. Non-Danish speaking patients and patients with an ethnic minority background were considered vulnerable by 74 and 84% of responding hospitals respectively. The corresponding percentages were lower for municipalities, 58 and 61% respectively. These percentages must also be interpreted with caution as some respondents were reluctant to stereotype by certain vulnerability parameters; and would rather do an individual assessment of needs and provide individualized healthcare services. The provision of interpreter services in hospitals reached 100% whereas it was only 84% for municipalities. The full coverage in hospitals can be seen in the light of hospitals being legally required to provide interpretation [33], while municipalities are not subject to the section of the Danish Health Act about provision of interpreter services. Municipalities are solely obliged to provide secondary prevention care equal to that of hospitals. The section about interpretation in the Danish Health Act is the only law that explicitly states mandatory use of interpreters specifically aimed at the public hospitals, medical specialists and general practitioners, thereby leaving a gap in other health services outside of these health facilities regarding the provision of interpreter services.

A study in 2012 mapped the provision of interpreter services in 240 health services (48 emergency services, 48 mental health services, and 144 primary care services) across 16 different European countries and found that 42% of the services did not provide any form of interpretation. Countries that tended to have higher provision of interpreter services had policies and regulations concerning cost of interpretation [36]. It is therefore fair to conclude that legislation on interpretation plays a significant role in the provision. The importance of having a legal framework for language in access to healthcare services has been discussed in the US, and that it should not an impediment to health [37]. Although the Danish and US healthcare systems are not directly comparable, the essence of the message is applicable. The differentiation in the Danish law between hospitals and municipalities seems to be reflected in the provision of interpreter services, albeit it is the very same Danish Health Act that sets out the obligations for shared responsibilities for CR. This has the potential to increase inequality in access for migrants to CR between the two sectors.

An aim of the involvement of municipalities in CR was to achieve greater equality in health and increase the inclusion of socially vulnerable patients according to WHO’s vision of “Health in All Policies” [8]. However, it seems that the inclusion of migrants in CR is inadequate.

Another interesting finding of our study was the very limited availability of multilingual information material reported to be offered by 6 hospitals, mainly concentrated at university hospitals, and hospitals with catchment areas with higher densities of migrants, and only in one municipality. This runs contrary to the findings of a review by Coulter et al. [27] showing major benefits for patient information material.

#### Strengths and limitations

The study was not designed specifically to identify migrants, hence this information was extrapolated from information related to non-Danish/limited Danish speakers and ethnic minorities. Ethnic minority does not in all cases mean a migrant, hence there is a small risk the survey overestimated the number of sites providing CR to migrants. However, given the available data we feel this paper gives a unique insight into existing structural aspects of CR related to migrants. We recommend future studies base survey design on official definitions of migrants.

A strength in this survey design is that the combination of close-ended and open-ended questions made it possible to solicit additional information to gain a more detailed understanding of the quantitative data.

The open-ended answers also demonstrated that items did not always capture the aimed for information, which could be considered as a weakness in the design. Because all data was self-reported, there is a risk that respondents may have reported answers that reflect a greater compliance with the guidelines which might entail information bias that tended to lead to more positive responses.

Another limitation is that we were only able to obtain information on provision of CR from 96% of the municipalities in contrast to 100% of the hospitals. This could potentially underestimate the results from the municipalities. Moreover, it was only possible to collect information on interpreter services, multilingual information material and socially vulnerable patients from hospitals and municipalities that offered socially differentiated CR, leading again to a potential underestimation of our results. New surveys will be conducted in 2018, and we plan to include the same items, and this time all hospitals and municipalities are included in order to follow the development on this area.

## Conclusion

In this descriptive study on migrants’ access to CR in hospitals and municipalities, we found two barriers to access. Firstly, not all hospitals provided CR core components for non-Danish speaking patients. Secondly, there was not full coverage of interpreter services in municipalities. Furthermore, only a few hospitals and municipalities provided multilingual information material. The observed inequalities in access to CR call for increased awareness in order to ensure effective treatment and prevention for migrants. There seems to be a need for more education and guidance for health professionals on the encounter with patients with a migrant background.

Lastly, there is a need for further studies to evaluate the actual scale of the problem; and to better understand why CR services are not fully accessible for migrants.

## Additional file

Additional file 1: **Table S1.** The table shows general provision of core components of cardiac rehabilitation and provision of CR for migrants at Danish hospitals. (DOCX 16 kb)

## Abbreviations

CR: Cardiac rehabiliation
DHRD: Danish Cardiac Rehabiliation Database
IHD: Ischemic heart disease
WHO: World Health Organization
