# Supplementary material for: Access to cardiac rehabilitation and the role of language barriers in the provision of cardiac rehabilitation to migrants
Source: BMC Health Serv Res. 2019 Apr 11;19:223. doi: 10.1186/s12913-019-4041-1 (PMC6458700; doi:10.1186/s12913-019-4041-1)
Supplement: Supplementary file 1 — Table S1. The table shows general provision of core components of cardiac rehabilitation and provision of CR for migrants at Danish hospitals. (DOCX 16 kb) [file 12913_2019_4041_MOESM1_ESM.docx]

Additional file 1: **Table S1.** General provision of core components of cardiac rehabilitation and provision of CR for migrants at Danish hospitals.

|  | 2013 | |  | 2015 | |
| --- | --- | --- | --- | --- | --- |
| Core components | Provision  N=36 | Provision for  migrants |  | Provision  N=35 | Provision for  migrants |
| Exercise training | 100%  (36/36) | 92%  (33/36) |  | 97%  (34/35) | 91%  31/34 |
| Patient education | 97%  (35/36) | 71%  (25/35) |  | 97%  34/35 | 74%  25/34 |
| Psychosocial support | 89%  (32/36) | 94%  (30/32) |  | 89%  31/35 | 94%  29/31 |
| Smoking cessation support | 94%  (34/36) | 82%  (28/34) |  | 92%  32/35 | 72%  23/32 |
| Nutritional counselling | 97%  (35/36) | 94%  (33/35) |  | 94%  33/35 | 94%  31/33 |
